# From liquid biopsy to spatial liquid biopsy: extracellular vesicles as molecular maps of cancer metastasis

**DOI:** 10.3389/fonc.2026.1940169

**Published:** 2026-10-20

**Authors:** Masato Mitsuhashi

**Affiliations:** NanoSomiX, Inc., Aliso Viejo, CA, United States

**Keywords:** biomarkers, blood test, cancer metastasis, extracellular vesicles, liquid biopsy, precision medicine

## Abstract

Cancer metastasis remains the leading cause of cancer-related mortality, yet current liquid biopsy approaches provide limited information regarding the anatomical distribution of metastatic disease. Circulating tumor DNA, circulating tumor cells, and extracellular vesicle (EV)-based assays have substantially advanced molecular characterization of cancer but generally do not reveal the tissue sites where metastatic progression is occurring. Here, we introduce the concept of Spatial Liquid Biopsy, a proposed next-generation framework that utilizes circulating tumor-associated EVs as molecular maps of metastatic disease. We hypothesize that EVs released from metastatic lesions may carry both tumor-associated signatures and tissue-associated extracellular proteins reflecting interactions with the local metastatic microenvironment. By integrating tumor-associated EV enrichment with multiplex profiling of organ-associated surface markers, Spatial Liquid Biopsy seeks to identify composite tumor-associated/organ-associated EV signatures that may provide greater spatial specificity than either marker alone. If experimentally and clinically validated, these signatures may enable minimally invasive inference of metastatic organ involvement and longitudinal assessment of metastatic disease evolution. This framework represents a conceptual expansion of liquid biopsy from detecting circulating evidence of malignancy toward generating spatial information that may complement imaging and guide personalized cancer surveillance and management.

## Introduction

Cancer metastasis remains the leading cause of cancer-related mortality and one of the greatest challenges in modern oncology (1). Although many primary tumors can be effectively managed through surgery, radiotherapy, and systemic therapies, metastatic disease remains largely incurable and is responsible for approximately 90% of cancer-related deaths (2).

Assessment of metastatic disease currently relies on a combination of anatomical imaging, pathological confirmation, and blood-based biomarkers. Computed tomography (CT), magnetic resonance imaging (MRI), and positron emission tomography (PET) are the principal imaging modalities used to detect metastatic lesions and determine their anatomical distribution (3). Complementing these approaches, blood-based biomarkers—including serum tumor markers and liquid biopsy analytes such as circulating tumor DNA (ctDNA), circulating tumor cells (CTCs), and extracellular vesicles (EVs)—provide valuable information regarding tumor burden, molecular alterations, therapeutic response, and mechanisms of treatment resistance (4, 5). Among these analytes, EVs have attracted considerable interest because they not only reflect the molecular characteristics of their parental cells but also actively participate in intercellular communication during tumor progression and metastasis (6). Despite these advances, currently available circulating biomarkers generally provide limited information regarding the anatomical location of metastatic disease.

This limitation represents an important unmet clinical need. Micrometastatic lesions frequently remain undetectable until they become radiographically apparent, and no routinely available blood-based assay can reliably identify the organ in which metastatic progression is occurring (7). Consequently, surveillance of patients at risk for metastatic recurrence relies heavily on repeated imaging studies, increasing healthcare costs and cumulative radiation exposure and potentially delaying recognition of emerging metastatic disease (7).

We propose that the next evolution of liquid biopsy should extend beyond the detection and molecular characterization of tumor-associated biomarkers to include the extraction of spatial information encoded within circulating EVs. We introduce the concept of Spatial Liquid Biopsy, defined as the use of organ-associated extracellular proteins detected on tumor-associated EV populations to infer the anatomical location of metastatic disease from a blood sample. We hypothesize that EVs released within metastatic lesions may display composite surface signatures comprising tumor-associated markers together with extracellular proteins associated with the local metastatic microenvironment. By first enriching EV populations using tumor-associated surface markers and subsequently interrogating them for organ-associated extracellular proteins, Spatial Liquid Biopsy seeks to achieve spatial specificity through the combination of these two biological dimensions rather than through either marker alone. This approach could enable minimally invasive assessment of metastatic progression together with inference of the most likely sites of metastatic involvement. Although this concept requires rigorous experimental and clinical validation, it provides a biologically testable framework that could complement conventional imaging and expand the scope of liquid biopsy in precision oncology. Conventional liquid biopsy primarily asks, “Is cancer present?” Spatial Liquid Biopsy seeks to address the additional question, “Where is the cancer?”.

## Conceptual framework

### From conventional liquid biopsy to spatial liquid biopsy

Cancer-derived EVs provide a circulating molecular window into tumor biology. Beyond serving as biomarkers, EVs actively participate in intercellular communication during tumor progression and metastasis by transferring proteins, lipids, and nucleic acids between cancer cells and the surrounding microenvironment (5, 6). Because the molecular composition of EVs largely reflects that of their parental cells, they have emerged as promising candidates for minimally invasive cancer diagnostics (5, 8, 9). When tumor cells express abundant cell-surface proteins that are incorporated into EV membranes, these proteins can be exploited to enrich tumor-associated EV populations from peripheral blood by immunocapture methods. Representative candidate capture markers include epithelial cell adhesion molecule (EpCAM), mucin 1 (MUC1), and epidermal growth factor receptor (EGFR) for lung cancer; EpCAM, human epidermal growth factor receptor 2 (HER2), and MUC1 for breast cancer; and EpCAM and carcinoembryonic antigen (CEA) for colorectal cancer, as shown in **Table 1**. Although these are well-established tumor-associated surface antigens, they may also be expressed by normal epithelial cells, making it difficult to distinguish tumor-derived EVs from the abundant background of circulating EVs released by healthy tissues (4, 6, 8, 9). This limitation presents an important challenge for the development of robust blood-based assays for monitoring metastatic disease.

**Table 1:** Representative candidate markers.

Purpose	Representative surface markers	Rationale
Lung cancer EV enrichment	EpCAM, MUC1, EGFR	Frequently expressed on lung epithelial tumors
Breast cancer EV enrichment	EpCAM, HER2, MUC1	Tumor-associated epithelial markers
Colorectal cancer EV enrichment	EpCAM, CEA	Common epithelial tumor markers
Brain metastasis	BCAN, NCAN	Brain-associated extracellular proteins
Liver metastasis	LECT2, SPP2	Liver- associated extracellular proteins
Bone metastasis	ALPL, BSP	Bone-associated extracellular proteins
Lung metastasis	SFTPA1, SFTPC	Lung associated extracellular proteins

We propose an alternative conceptual framework. Rather than relying exclusively on highly cancer-specific EV markers, tumor-associated EV populations may also encode spatial information reflecting the metastatic tissue environment in which they are generated. During metastatic colonization, tumor cells undergo continuous molecular and cellular interactions with extracellular matrix components, stromal cells, endothelial cells, immune cells, and organ-specific resident cells. These include, for example, Kupffer cells and hepatic stellate cells in the liver; astrocytes and microglia in the brain; and osteoblasts, osteoclasts, and bone marrow stromal cells in bone. Through reciprocal signaling involving cytokines, growth factors, extracellular matrix interactions, and EV-mediated communication, these local cellular environments can influence the molecular phenotype of metastatic tumors and the EVs they release (5, 10). In addition, we hypothesize that extracellular proteins present within these organ-specific metastatic microenvironments may become associated with the surface of locally generated tumor-derived EVs. Consequently, EVs entering the circulation from metastatic lesions may carry composite surface signatures consisting of tumor-associated markers together with organ-associated extracellular proteins reflective of the metastatic microenvironment (**Figure 1**). As shown in **Table 1**, brevican (BCAN) and neurocan (NCAN) are representative candidate markers for brain; leukocyte cell-derived chemotaxin-2 (LECT2) and secreted phosphoprotein 2 (SPP2) for liver; alkaline phosphatase, liver/bone/kidney (ALPL) and bone sialoprotein (BSP) for bone; and surfactant-associated protein A1 (SFTPA1) and surfactant protein C (SFTPC) for lung. These candidates are well-established proteins associated with their respective tissues, supported by extensive expression and biological evidence in the literature.

**Figure 1:**
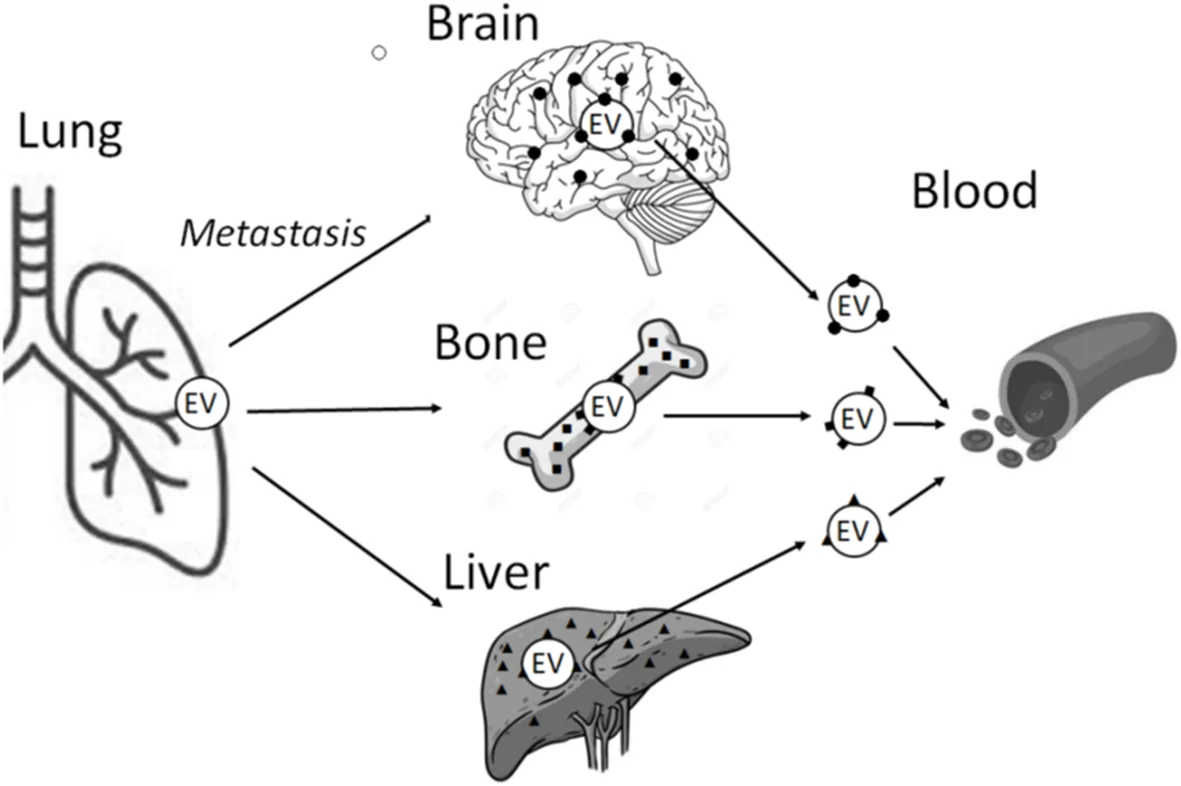
Illustration of the concept of spatial liquid biopsy using lung cancer as an example. Lung cancer cells release extracellular vesicles (EVs) from both the primary tumor and metastatic lesions in distant organs, including the brain, bone, and liver. We hypothesize that EVs released within metastatic lesions may display organ-associated extracellular proteins (●, ■, ▲) reflecting interactions with the local metastatic microenvironment, in addition to tumor-associated surface markers. Following entry into the circulation, EV populations are first enriched using tumor-associated surface markers and subsequently interrogated for organ-associated extracellular proteins. The resulting tumor-associated/organ-associated double-positive EV populations represent the central analytical principle of Spatial Liquid Biopsy and may enable inference of metastatic sites from peripheral blood samples.

We define Spatial Liquid Biopsy as a liquid biopsy strategy that combines enrichment of tumor-associated EV populations with profiling of organ-associated extracellular proteins to infer the anatomical location of metastatic disease from a peripheral blood sample (**Figure 1**). Unlike conventional liquid biopsy, which primarily detects the presence or molecular characteristics of cancer, Spatial Liquid Biopsy seeks to provide information about where metastatic disease is occurring by exploiting organ-associated molecular signatures detected within tumor-associated EV populations.

For example, as illustrated in **Figure 1**, lung cancer cells that metastasize to the brain retain molecular features of the primary tumor while interacting with and adapting to the unique brain microenvironment. EVs released from these metastatic lesions may therefore carry both lung cancer-associated surface markers and brain-associated extracellular proteins (**Figure 1**●). Detection of brain-associated proteins within lung cancer marker-enriched EV populations in peripheral blood could therefore provide evidence consistent with brain metastatic involvement. The same principle could extend to other common metastatic sites, including bone (**Figure 1**■) and liver (**Figure 1**▲), provided that suitable organ-associated extracellular markers are identified and validated.

Importantly, the specificity of Spatial Liquid Biopsy does not depend on either the tumor-associated or organ-associated marker being individually specific. Tumor-associated markers such as EpCAM or MUC1 may also be expressed by normal epithelial tissues, while organ-associated proteins may not be absolutely restricted to a single organ. Rather, spatial specificity is expected to arise from the combination of these two biological dimensions. EVs released by normal tissues would not be expected to simultaneously exhibit a selected tumor-associated phenotype and an extracellular protein signature associated with a distant metastatic organ. Conversely, EVs released by the distant organ itself may carry organ-associated proteins but would not necessarily exhibit the selected tumor-associated phenotype. Spatial Liquid Biopsy therefore employs a two-dimensional strategy in which EV populations are first enriched using tumor-associated surface markers and then interrogated for organ-associated extracellular proteins. The resulting tumor-associated/organ-associated double-positive EV population represents the central analytical principle of Spatial Liquid Biopsy and is hypothesized to provide greater spatial specificity than either marker alone. If experimentally and clinically validated, this combinatorial approach could extend conventional liquid biopsy beyond detecting the presence of cancer to providing minimally invasive information regarding the anatomical distribution and longitudinal progression of metastatic disease.

## Evidence linking EV profiles to organ-specific metastasis

Several studies provide complementary, although indirect, support for the broader concept that EV protein composition can contain information related to organ-specific metastasis. Hoshino et al. demonstrated that tumor-derived exosomes from experimental models with different metastatic organotropism exhibit distinct surface integrin profiles (11). Exosomal integrins α6β4 and α6β1 were associated with lung tropism, whereas αvβ5 was associated with liver tropism, and these EVs preferentially interacted with resident cells in the corresponding target organs. Importantly, these integrins are transmembrane proteins expressed by tumor cells and carried by tumor-derived EVs. They represent determinants of metastatic organotropism rather than proteins derived from the metastatic organ or its microenvironment. Thus, this study demonstrates that tumor-derived EV surface composition can contain information related to organ-specific metastatic behavior, but it does not directly demonstrate the mechanism proposed in Spatial Liquid Biopsy.

Complementary evidence has been obtained from human circulating EVs. Li et al. compared plasma EV proteomes from patients with lung cancer with solitary liver metastasis, solitary brain metastasis, or locally advanced disease without distant metastasis (12). They identified distinct EV-associated protein profiles according to metastatic site and subsequently validated candidate proteins associated with brain or liver metastasis in a larger patient cohort. However, these investigators analyzed bulk plasma EVs rather than tumor-derived EVs specifically enriched using tumor-associated markers. Consequently, the cellular origin of the identified EV proteins cannot be assigned to the tumor, metastatic organ, or other host cells. Nevertheless, this study provides important human evidence that circulating EV protein profiles can contain information associated with the anatomical site of metastatic disease.

Additional experimental support comes from Nadeau et al., who characterized EVs released by breast cancer cell lines with distinct organotropic metastatic phenotypes involving lung, bone, liver, and brain (13). EV proteomic profiles differed according to the organotropic phenotype of the tumor cells. However, these EVs were collected from cultured tumor cells rather than from the circulation of animals or patients with established metastatic disease. This study therefore supports an association between tumor-cell organotropic phenotype and EV composition but does not demonstrate organ-associated proteins on circulating tumor-derived EVs.

Taken together, these studies support three relevant observations: (1) tumor-derived EVs can carry tumor-cell proteins associated with organ-specific metastatic tropism; (2) bulk circulating EV protein profiles in patients can differ according to the anatomical site of established metastasis; and (3) tumor cells with different organotropic metastatic phenotypes can release EVs with distinct proteomic profiles. However, to our knowledge, direct evidence that tumor-associated EVs specifically enriched from the circulation of patients with established metastases carry extracellular surface proteins associated with the metastatic organ or its local microenvironment remains limited. This knowledge gap provides a specific and experimentally testable basis for the Spatial Liquid Biopsy framework.

## Extracellular vesicles as molecular maps of metastatic disease

Simultaneous profiling of tumor-associated and organ-associated extracellular proteins on circulating EVs may enable construction of molecular maps of metastatic disease. For example, tumor-associated EV populations displaying bone-associated extracellular proteins may indicate skeletal metastasis, whereas those carrying liver-associated proteins may suggest hepatic involvement. Likewise, detection of brain-associated signatures within tumor-associated EV populations could provide evidence of central nervous system metastatic involvement. Integration of multiple organ-associated EV signatures may therefore enable blood-based inference of the anatomical distribution of metastatic disease (5, 10).

In principle, a patient with metastases involving multiple organs could exhibit multiple distinct tumor-associated EV subpopulations in the circulation, each reflecting a different metastatic site. For example, in a patient with simultaneous hepatic and osseous metastases, one subpopulation associated with the hepatic metastatic microenvironment could carry liver-associated proteins, whereas a separate subpopulation associated with the osseous metastatic microenvironment could carry bone-associated proteins. Thus, the proposed composite spatial profile refers to the coexistence of distinct organ-associated EV subpopulations in the same blood sample, rather than to individual EVs carrying markers from multiple metastatic organs. Multiplexed profiling of the organ-associated markers presented in **Table 1** could potentially distinguish and quantify these separate spatial EV signals.

Whether the relative abundance of individual spatial EV signals provides quantitative information regarding organ-specific metastatic burden represents an additional testable hypothesis. EV-associated signal magnitude may be influenced not only by tumor volume but also by EV release, vascular access, clearance, tumor biology, and characteristics of individual metastatic microenvironments. Therefore, signal magnitude should not be assumed to be directly proportional to metastatic burden. Establishing such a relationship would require correlation of individual organ-associated EV signals with independently determined disease burden, such as imaging-derived lesion number or volume. Longitudinal within-patient measurements may be particularly informative for determining whether changes in individual spatial EV signatures parallel progression or regression of disease at the corresponding anatomical sites.

Such an approach has the potential to extend liquid biopsy from a technology that primarily answers “Is cancer present?” to one capable of addressing an additional clinically actionable question: “Where is metastatic disease located?” Unlike conventional liquid biopsy, which focuses principally on detecting tumor-derived genetic or protein biomarkers, Spatial Liquid Biopsy seeks to infer the probable anatomical location of metastatic lesions by combining tumor-associated EV enrichment with organ-associated surface protein profiling.

Longitudinal analysis of organ-associated EV signatures may provide additional value for disease surveillance. Progressive or newly emerging organ-associated EV signals may indicate metastatic dissemination before lesions become radiographically detectable, potentially prompting targeted imaging or closer clinical monitoring. Conversely, declining organ-associated EV signatures following therapy may ultimately provide an early indication of treatment response. These potential applications will require prospective clinical validation. Spatial Liquid Biopsy is therefore envisioned as a complementary tool rather than a replacement for conventional imaging modalities, with the potential to support earlier detection, personalized surveillance, and more targeted use of diagnostic imaging (4, 7).

Development of Spatial Liquid Biopsy will require rigorous selection and validation of organ-associated EV surface markers, characterization of tumor-associated EV enrichment, and clinical studies correlating EV-derived spatial signatures with radiographic, pathological, and clinical outcomes. The candidate biomarkers presented in **Table 1** provide an immediately testable starting panel based on established tissue-expression patterns and biological evidence. If these predefined candidates do not provide sufficient discriminatory performance, unbiased proteomic approaches could subsequently be used to identify additional or complementary markers. Mechanistic studies will also be needed to determine how metastatic organ microenvironments contribute to the surface composition of tumor-associated EV populations. Despite these challenges, the potential for EVs to simultaneously reflect tumor-associated and metastatic-organ-associated information provides a biological rationale for developing blood-based technologies capable of monitoring metastatic disease in both spatial and temporal dimensions (5, 6, 10).

Collectively, these concepts suggest a potential expansion of oncology diagnostics from conventional liquid biopsy, which primarily detects circulating evidence of malignancy, toward Spatial Liquid Biopsy, in which EV-associated signatures may function as molecular maps providing information not only about the presence of metastatic disease but also about its probable anatomical distribution. If experimentally and clinically validated, this framework could improve the detection, surveillance, and management of metastatic cancer.

## Challenges and future directions

Spatial Liquid Biopsy is proposed as a conceptual framework and remains to be established through systematic experimental and clinical investigation. Its successful implementation will depend on several biological and technical considerations, including the abundance of tumor-associated EV populations in peripheral blood, the availability of robust tumor-associated EV capture markers, and the identification of organ-associated extracellular proteins that reliably reflect metastatic organ involvement. Because these factors are likely to vary among cancer types, metastatic sites, disease stages, treatment status, and individual patients, optimization of biomarker selection and assay design will be essential.

A central challenge will be the identification and validation of organ-associated extracellular proteins that provide sufficient discriminatory information for metastatic site inference. Absolute organ specificity of individual proteins may not be necessary because the central analytical principle of Spatial Liquid Biopsy is based on the combination of tumor-associated capture and organ-associated detection. Nevertheless, individual organ-associated proteins may have insufficient sensitivity or specificity, and multiplex analysis incorporating complementary markers may provide greater robustness than reliance on a single marker. Likewise, combinations of tumor-associated capture markers may be required to accommodate molecular heterogeneity among cancers and their metastatic subtypes. The candidate tumor-associated and organ-associated proteins presented in **Table 1** provide an immediately testable starting panel; unbiased proteomic discovery could subsequently be used to identify additional or complementary markers if predefined candidates provide insufficient discriminatory performance.

The proposed framework also raises important biological questions regarding the mechanisms by which organ-associated extracellular proteins become detectable on circulating tumor-associated EV populations. Future mechanistic studies should investigate how interactions between metastatic cancer cells and the local microenvironment—including stromal cells, endothelial cells, immune cells, extracellular matrix components, and organ-specific resident cells—influence EV composition and how extracellular proteins present within the metastatic microenvironment may become associated with tumor-derived EV surfaces. Improved understanding of these processes may not only strengthen the biological foundation of Spatial Liquid Biopsy but also provide new insights into metastatic niche biology.

Technological advances are expected to facilitate development of this approach. Continued improvements in EV enrichment, high-affinity antibody engineering, multiplex protein detection, ultrasensitive immunoassays, and single-EV analytical technologies should enhance the ability to characterize complex EV surface phenotypes from small blood samples. Integration of these technologies with computational approaches, including machine learning for multidimensional biomarker analysis, may further improve discrimination among metastatic sites and interpretation of composite spatial EV signatures.

A logical pathway for clinical translation would involve staged validation. Initial proof-of-concept studies could evaluate the predefined candidate markers presented in **Table 1** using archived plasma specimens from patients with anatomically confirmed metastatic disease. Lung cancer represents a particularly suitable initial model, as illustrated in **Figure 1**, because it frequently metastasizes to anatomically distinct sites including brain, bone, and liver. Patients with a single dominant metastatic site would provide informative initial cohorts for determining whether tumor-associated/organ-associated EV signatures discriminate among these sites. Following analytical and retrospective clinical validation of individual spatial signatures, studies could extend to patients with multi-organ metastatic disease to determine whether distinct organ-associated EV subpopulations can be detected concurrently. Subsequent prospective and longitudinal studies would be required to determine whether emerging or changing spatial EV signatures correlate with radiographic findings, pathological confirmation, treatment response, and clinical outcomes.

An additional challenge will be determining whether the abundance of individual spatial EV signatures provides quantitative information regarding organ-specific metastatic burden. EV-associated signal magnitude may reflect not only tumor volume but also EV production and release, vascular access, clearance, treatment effects, and characteristics of individual metastatic microenvironments. Therefore, quantitative interpretation will require correlation with independently determined measures of organ-specific disease burden, such as imaging-derived lesion number or volume. Longitudinal within-patient measurements may be particularly informative for determining whether changes in individual spatial EV signatures parallel progression or regression of disease at corresponding metastatic sites.

Although considerable biological, technical, and clinical validation remains necessary, the proposed framework establishes a testable foundation for extending the capabilities of liquid biopsy. Rather than simply detecting circulating evidence of malignancy, Spatial Liquid Biopsy seeks to extract spatial information from composite tumor-associated/organ-associated EV signatures, enabling minimally invasive inference of the probable anatomical distribution of metastatic disease. If successfully validated, this approach could complement conventional imaging, improve metastatic surveillance, facilitate earlier clinical intervention, and expand the role of liquid biopsy in precision oncology.
